# Effects of changing development patterns and ignition locations within Central Texas

**DOI:** 10.1371/journal.pone.0211454

**Published:** 2019-02-07

**Authors:** William Mobley

**Affiliations:** Department of Marine Sciences, Texas A&M University at Galveston, Galveston, Texas, United States of America; Rochester Institute of Technology, UNITED STATES

## Abstract

Researchers have a limited understanding of the interactions between development patterns and Ignition Probability. This study explores the variation in Ignition Probability as a result of differing development patterns. Based on LANDFIRE datasets, development changes were mapped for two sets of years (2001 and 2012) and the relationship between development and Ignition Probability was assessed. The study area covered the two adjacent counties, Bastrop and Travis located in Texas, USA. These two counties have a high potential for wildfire, and due to expanding development have high vulnerability. Expanding lateral development was organized into one of five categories: infill, radial, isolated, clustered, and linear. The Maximum Entropy algorithm predicted the spatial distribution of ignition probabilities based on several physical and land use characteristics coupled with historic ignition locations. Variation in Ignition Probability was assessed for each category of development using two-way ANOVA’s and *post hoc* analysis. Ignition Probability maps indicated a fair sensitivity (Area Under the Curve: 0.77–0.78), suggesting that the spatial configuration of development patterns influenced wildfire ignition. Analyses found that isolated, clustered, and linear outlying development patterns had higher Ignition Probability than infill and radial development, and that fire probabilities fell along a development gradient. This trend between the development gradient and ignition probabilities should be used to direct land use planning to reduce fire risk.

## Introduction

Seven of the most destructive fire seasons in the U.S. have occurred in the last 15 years, four of which have burned over 9 million acres. The 2015 fire season was the first fire season on record to burn over 10 million acres, and 12,306 structures were burned during the 2017 fire season [1]. Federal agencies are having to spend more each year to suppress wildfires [2]. Taking inflation into account, suppression expenditures have quadrupled since 1985 (2015 $1.9 billion). Despite the increased spending, wildfires continue to burn thousands of structures yearly [3]. These structural losses from increased acres burned, are caused by several factors including: a high percentage of anthropogenic ignitions [4], wildland urban interface (WUI) expansion [5], and climate change [6, 7].

Within the United States, the creation of the Federal Housing Administration has reduced constraints for financing the development of single family homes [8], which in turn has stimulated the growth of single family development into increasingly suburban and exurban areas [9]. Previously these areas were often wildlands [10]. Areas of developed lands surrounded by wildland vegetation (i.e. WUI) are vulnerable to wildfire [11], and understanding how development patterns influence ignition probabilities in the WUI will help planners and policymakers create more resilient communities.

Creating a fire resilient community requires understanding the probability of a disturbance and the factors that shift this probability. Ignition Probability (IP) models identify where on a landscape a fire might ignite by predicting the likelihood of wildfire ignitions across space. The outcome is a raster dataset that denotes, for each cell, its probability of ignition given a set of spatially explicit environmental predictors. Ignition datasets are presence only and identify the location of ignitions that have occurred, however, some ignitions may go undetected. IP models have evolved from species distribution models [12], which are also implemented using presence-only datasets. By adopting a similar approach, IP modeling can empirically predict a continuous raster where a fire is likely to begin [13].

### Wildland urban interface and development patterns

During the 1980’s and 1990’s research has identified the WUI as a subject area in need of special examination in terms of mitigating wildfire risk [14]. Research has illustrated that fires located within WUI areas were more likely to burn structures at a higher rate than non-WUI located fires [15]. The WUI makes up a significant proportion of landcover in the U.S., approximately 10% in 2010 and has been expanding rapidly in recent decades [15]. An even larger proportion of homes (33.5%, [5]) are located adjacent to the WUI. The WUI’s development composition adjacent to large fuel loads cause structures within these areas to be at high risk from wildfire [15]. The vulnerability of the WUI, has led researchers to map and understand the risk associated with the WUI [16]. Risk trends show that lower and intermediate housing densities are more likely to burn than those at higher densities [17]. However, complicating the analysis, the higher densities referenced were in the urban core, where vegetation and fuels are often highly fragmented by asphalt and concrete. Other research suggests that buildings within the WUI may increase the chance of those structures burning [18]. Although mapping the WUI helps identify areas vulnerable to wildfire, the form of development patterns within the WUI vary greatly.

Most development occurring in the WUI is a result of urban sprawl. Sprawl definitions vary depending on the study and the question addressed [9, 19, 20], but consistently are focused on low density development [9, 21], which is frequently expressed as the degree of sprawl [20]. Initially, urban development described cities as monocentric, where the city has one core business area and development extends outwards from the core. This suggests that three kinds of development patterns exist: vertical growth, infill growth, and lateral growth [22]. Contemporary sprawl metrics still resemble these initial patterns [23, 24]. Vertical growth replaces single family homes with higher density larger structures. Infill growth occurs on vacant land, either lots or open space in settled areas.

Initially, cities were thought to develop as lateral growth rings extending from the central business district [22]; however, this description of rings fails to account for multi-nuclearity or sector type development [25]. Due to the complexity of urban development, more recent research has focused on expanding the characterization of lateral growth. For example, differentiating between radial growth, where development occurs in an area surrounded by less than 40% development, and outlying growth, where new development occurs away from previous development [26]. Outlying growth is further characterized by three additional growth patterns including isolated, linear branch, and new clustered development [26]. Isolated outlying growth consists of small patches of development separate from other development, which has often been referred to this as “leap frog growth” [9, 23, 27]. Outlying linear branch growth occurs on transportation corridors such as roads and rail lines, for instance, strips of commercial development along a road [9, 20]. Outlying clustered development consists of multiple structures (i.e. subdivisions or industrial developments), which are larger than those found in isolated development.

Lateral growth patterns have also been measured in various ways. For example, measuring the degree of changes in development between one year to the next, spatial location of new urban development and the threshold of the percentage of surrounding urban (40%) can predict whether an area would be infill or radial [26]. Other research has used more complex metrics such as contagion [21], fractal dimensions [28] and interspersion and juxtaposition indices to estimate lateral sprawl. Contagion estimates how urban patches are dispersed [16]. Fractal dimension helps estimate the position of a city on a scale between the states of completely compact and completely dispersed [28].

### Ignition probability

Ignition is the initial step in any fire. While only a limited number of ignitions develop into large fires, yet each ignition has the potential to grow larger and become destructive. Ignition based models use historic ignition or fire points to gain a better understanding of the spatial nature of wildfire. The point data allows for a variety of methods to understand the nature of the data. Points can be used to understand the landscape’s fire regime, by associating ignition rates globally [29] or used to create a local IP raster [30]. Overall, these analyses allow researchers to understand the fire regime and fire frequency on a landscape and predict deviations from historic patterns across time or space [12].

While research has identified WUI areas as vulnerable, few studies have assessed how development of the landscape interacts with IP. One study indicated differences in fire arrangements between urban patch densities and forest fragmentations [31], but the study was cross-sectional and did not specifically identify which development patterns were the riskiest. A second, broader cross-sectional study found a positive correlation between fire frequency and population [29]. Of the studies focusing on lateral development, evidence suggests that outlying development results in a higher fire frequency than infill [32], however, this study only assessed a single landscape at one point in time. Another body of research focused on the likelihood of damage to structures by using either statistical or machine learning algorithms to estimate structural damage. Results indicate that housing density influenced the likelihood of a home burning [33, 34]. Defensible space and low slopes also help reduce structure damage[35], while small, high-density clusters of development or low density structures were more at risk for burning than other development types [33].

The primary objective of this study was to determine whether different categories of development had different anthropogenic IP distributions. Specifically, this study hypothesizes that ignition probabilities will differ based on the nearest development type. To meet this objective this study categorized new development patterns and then quantified how IP surrounding specific development types differed between two sets of years. More specifically, using two-way ANOVA’s and *post hoc* analyses, this study determines and orders by types of lateral development. The paper then discusses these findings in context of previous studies and then summarizes the conclusions of the paper.

## Methods

### Study area

**Fig 1:**
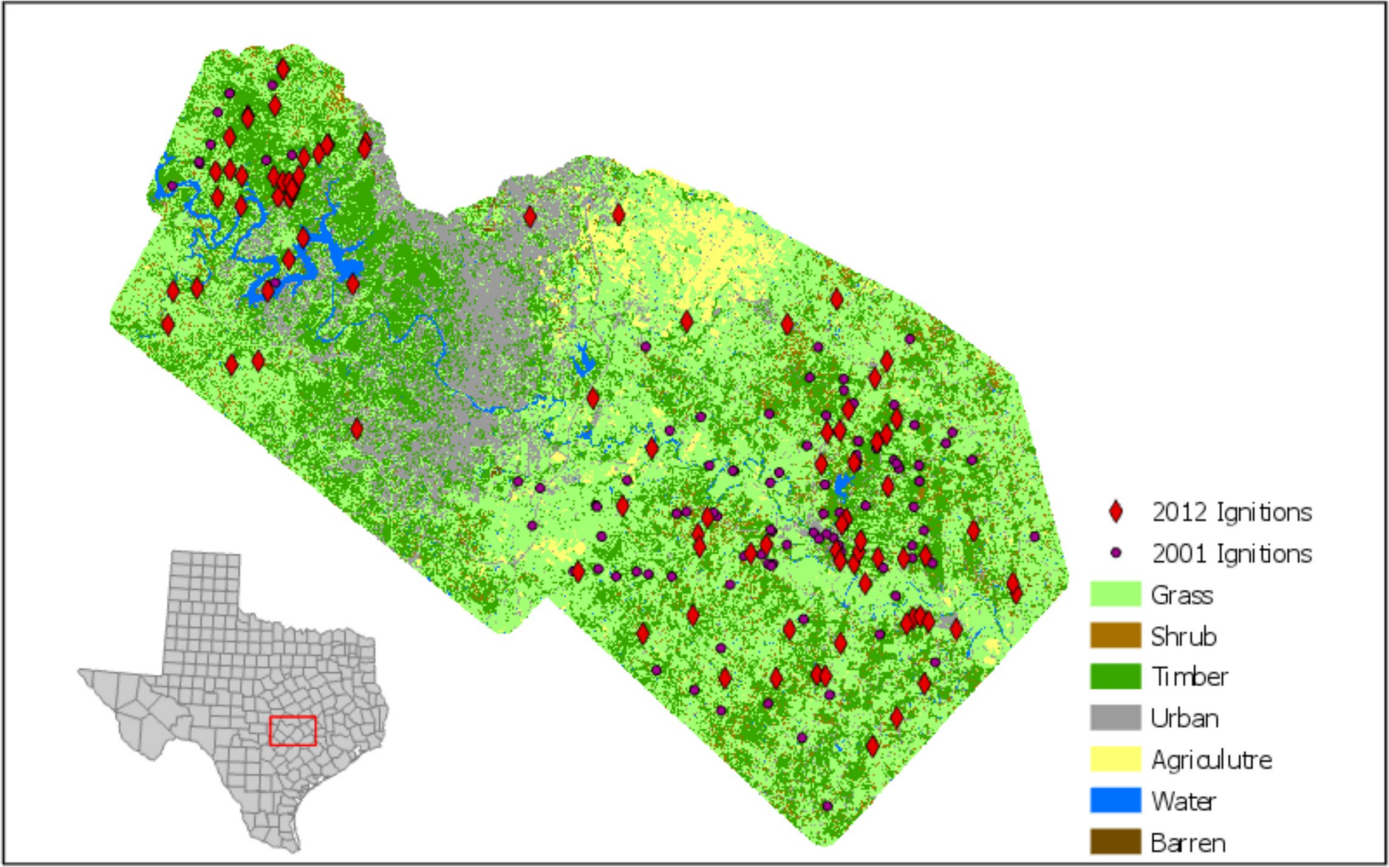
Study area of Bastrop and Travis County. Fuel types are aggregated to more generalized categories.

The study area encompasses Travis and Bastrop Counties in central Texas (Fig 1). Travis County consists of 356,000 hectares, and Bastrop county is 310,000 hectares. Travis County is a large (1.02 million people in 2010), fast-growing community (26% growth over 10 years; [36]). This population growth has led to an increase in development (21,214 ha 2001–2012 [37]). Bastrop County’s smaller population (74,000) is a stark contrast to Travis County. Bastrop County has a similar growth rate over the 10-year study period (28% growth; [36]). Because of the smaller population size, Bastrop County has experienced less development growth (1,343 ha 2001–2012). Travis County is higher in elevation (433m peak) compared to Bastrop County.

In 2012, much of the vegetation that existed within the study area was grass cover (229,000 ha), followed by forest (156,389 ha) and shrub lands (17,174 ha [26]). Grasslands have increased the most (88,998 ha since 2001) as a result of conversion to pasture lands. The largest changes to the landscape have occurred through tree cover growth (42,000 ha), which has occurred primarily in Bastrop County.

Bastrop and Travis counties have had approximately 3,000 ignitions from 1999–2015 [38], most of which started in Forests. The fires within the study area are primarily small (77% are half a hectare or smaller); however, the study area has also had eleven fires which burned more than a 1,000 acres. Most recent fires have occurred in Bastrop County, which experienced over 2000 fires between 2010 and 2014. The extensive growth of communities combined with the fires that have occurred in the area, makes these counties an optimal study area to understand the effects of development on the fire landscape. The time period for the study focuses on two sub-groups of years (1999–2003 & 2010–2014). From this point forward, the 1999–2003 time period will be referred to as the 2001 model and the 2010–2014 time period will be referred to as the 2012 model.

### Development categories

**Fig 2:**
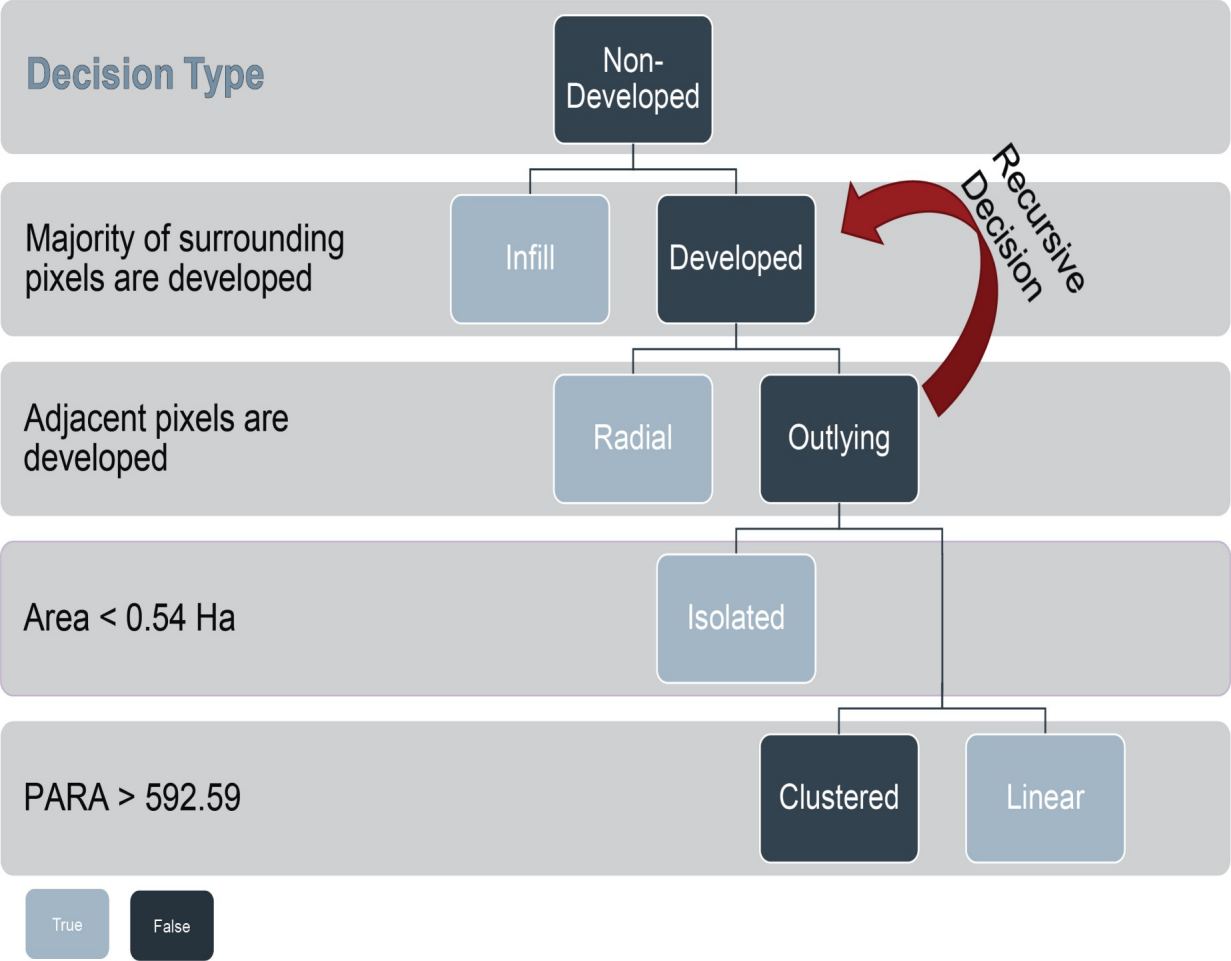
Categorization flowchart for developed lands. This includes the rules used to identify patch, perforated and interior wildlands.

Because landscapes are dynamic, many urban studies focus on lateral growth. This study categorized development using a decision tree approach (Fig 2), where each step in the decision tree separates a development type. This separation started with infill followed with radial and then sub-categorized three outlying development patterns: isolated, clustered, and linear. Isolated growth consists of small patches of development separate from other development. Linear growth occurs on transportation corridors such as roads and rail lines while new clustered development consists of multiple structures. This study’s approach was modeled after [26] but was not identical. The main change for this study, is the use of a recursive iteration on new development patches that are adjacent to previous development. These pixels are defined as radial development categories, this approach allows for larger developments to be classified as radial beyond adjacent pixels.

**Fig 3:**
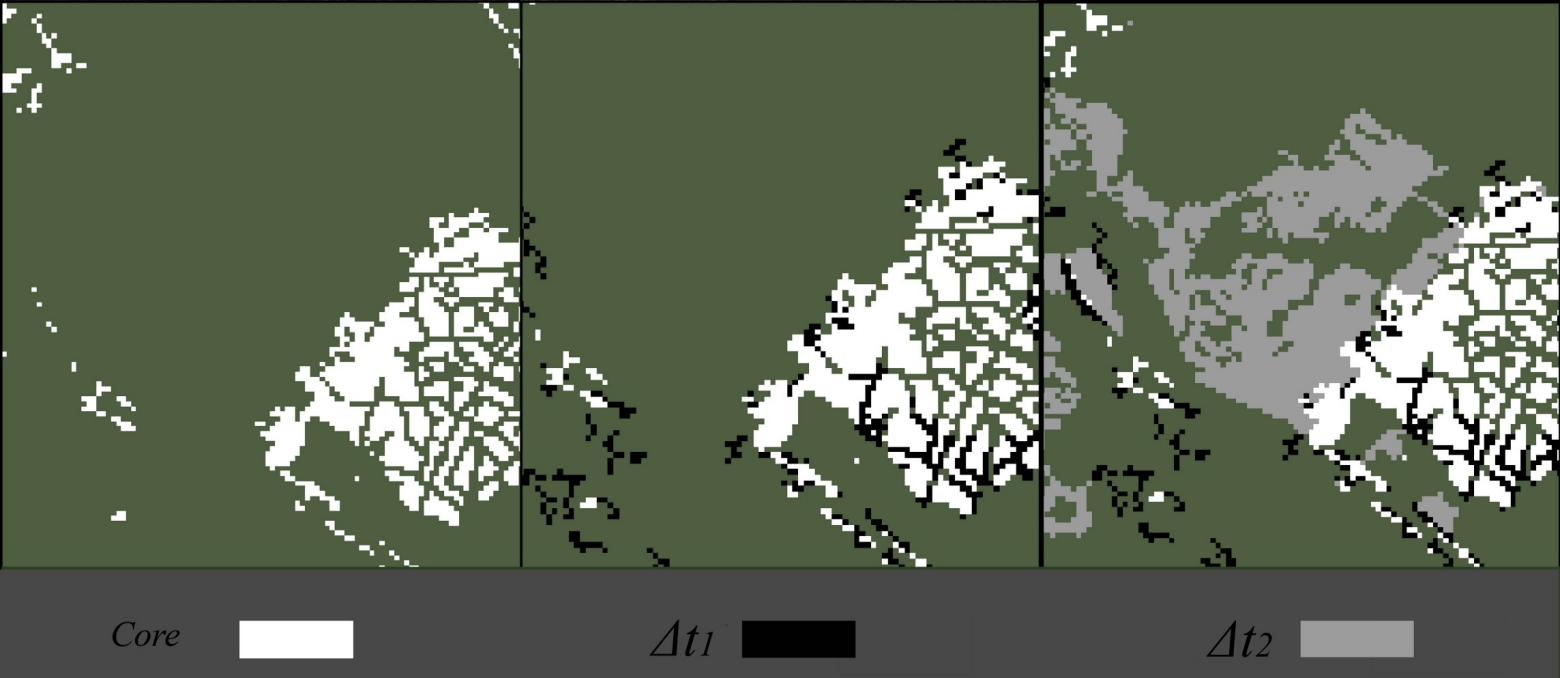
Generations of development. Note the transitions in development between 1996–2001 and 2001–2012.

The study focused on two time periods where new development occurred, therefore, three years of land cover (1992, 2001, and 2012) were used to define the two generations of development (Fig 3). Each generation was defined as development that had not occurred previously. For example, the Δt_1_ occurred on the 2001 landscape but not the 1992 landscape, while the Δt_2_ occurred on the 2012 landscape but not the 2001 landscape. The land cover data came from two different sources, National Land Cover Dataset (NLCD) [39] and LANDFIRE [37], both of which had a grain size of 30m. Development from 2001 and 2012 were calculated from LANDFIRE, ensuring that the development patterns match with the fire simulations. LANDFIRE does not publish a land cover dataset pre-2001, so NLCD’s 1992 retrofit dataset was used as a baseline for previous development. A qualitative exploratory assessment of NLCD’s 2001 dataset against LANDFIRE’s 2001 dataset showed little difference between land covers, suggesting that using the NLCD 1992 would not create inaccuracies in the assessment.

The NLCD land cover dataset provides three classifications for developed area, including high, medium, and low intensity development, while the LANDFIRE dataset provides one. Within the NLCD dataset the three development intensities were aggregated into a single class (developed), while in both datasets the vegetation classes were aggregated into a single class (wildland). Once the initial developed and wildland areas were classified, new development for each year was identified. Any development that existed prior to 2001 was classified as core development. New developed pixels were categorized as wildland pixels that transitioned to urban between years. Any development that existed prior to 1992 was classified as previous development.

Categorizing lateral development patterns was performed for both time periods beginning with 2001 and followed by 2012. First, infill was identified as new development that occurred in areas with > = 60% developed pixels. The initial infill pattern was generated using a square moving window size of 1920 m (64 pixels) per side. The window size was derived from a lacunarity analysis of new development in the study area [40].

After infill was identified, radial development was categorized. Pixels were categorized as radial development if it was adjacent to a previously developed pixel. The radial decision process was iterated until no more pixels were categorized as radial. All newly developed pixels, that were not categorized as either infill or radial were categorized as outlying development.

Outlying development occurs in three categories: isolated, clustered, or linear. Patches of newly developed pixels were used instead of individual pixels to identify the appropriate class. The Patch Area and Perimeter Area Ratio (PARA) were calculated for each patch. PARA measures the perimeter of the patch over the area [41]:

$$PARA=\frac{p}{a}$$ (1)

Where *p* is the perimeter of a patch and *a* is the area of that same patch.

Since linear and clustered development have larger areas, isolated development was the first to be classified of the three outlying patterns. Isolated development patterns consist of a small number of buildings, often individual structures or with detached sheds, in an area. Because NLCD resolution is not fine enough to determine individual structures, the study assumed that the number of structures was correlated with patch size. Isolated patches were assessed and categorized using the distribution of patch areas for new outlying development. Any new outlying development patch that had an area below the median value (0.54 hectare) was classified as isolated. The median value was used due to a flooring effect of the distribution of patch areas. Those developments, that were not classified as isolated were then classified based on their linearity using PARA.

Any new outlying-development patch with a PARA that exceeded median value (592.59 m^-1^) was designated as linear development, while any patches below that value were designated as clustered development. Using the PARA metric is not without its downside; the PARA metric struggles to differentiate between linear patches and patches with complex edges [41]. However, because the PARA metric was used solely on new clustered development, the edges were not complex and an inspection of the two classifications showed the threshold to be acceptable.

Once all development types were added, the aggregate median center was calculated for each development category. The median center addresses where both Δt_1_ and Δt_2_ are primarily developing as compared to the previous development. Development patterns, such as infill, should be developing closer to previous development, while outlying patterns should occur further away.

### IP model

The MaxEnt Software v3.3 was used to predict IP. Previous research has demonstrated that MaxEnt provides accurate results for predicting the occurrence of natural hazards [12, 42]. MaxEnt predicts the probability of an event based on point observations of occurrence (i.e., the dependent variable) and background environmental data (i.e., independent variables) [43]. The independent variables may be continuous, categorical, or binomial. IP is calculated by maximizing entropy in covariate space [44] and also takes into account non-linearities and interactions between variables [45]. Machine learning algorithms, such as MaxEnt, are more robust when dealing with issues caused by statistical assumptions. For instance, MaxEnt provides a stable model in the face of highly correlated independent variables [44]. The dependent variable, fire events, was sourced from the historic fire datasets provided by Texas Forest Service and USGS and consisted of all documented wildfires between 1999 and 2014. The output of MaxEnt is the map of the probability of occurrence of the dependent variable (here, IP).

### Variables

#### Dependent

The IP model was based on anthropogenic ignition locations gathered from local fire departments, the Texas Forest Service, and federal agencies [38]. This dataset covers fires from 1999–2015 (see Table 1). Of the 3,027 fires that were recorded during this time, 98% were caused by humans and over half of the fires were caused by debris burning. On average fires between 2010 and 2014 were larger than those between 1999 and 2003, burning 29 and 11 hectares respectively. Part of this high average is due to the Bastrop complex which burned 14,000 Ha. The Bastrop Complex fire was driven by extreme weather conditions during a long drought in 2011 [46]. A preliminary model suggested that a smaller subset of years (5 years) produced a more sensitive model when compared to a larger subset of years. This is likely because ignition’s drivers (e.g. land cover) shift over time, and studies with a larger temporal range cannot take this into account [47]. A five-year sample surrounding the date of each land cover classification helped reduce the model’s potential for errors. Therefore, two subsets were created from the initial dataset for the two study periods (1999–2003 and 2010–2014), which yielded sample sizes of n = 123 and n = 1156, respectively. The earlier ignition sample (2001) is much smaller than the more recent (2012) group because Texas fire departments did not keep records of fire locations prior to 2005. Initially, the 2012 model was run twice, with and without the extra Texas Fire Departments data. Using Area Under the Curve (AUC), the model with all ignitions included produced a better sensitivity as compared with the reduced ignition 2012 model, however, mean probabilities by development type were lower for the reduced ignition model. Because of this, the reduced ignition 2012 model was used as findings would be more conservative. Since each ignition group was centered on specific land cover years, minimal change was assumed in socio-economic and landscape variables.

**Table 1:** Ignitions within the study area separated by year.

Year	1999	2000	2001	2002	2003	2004	2005	2006	2007	2008	2009	2010	2011	2012	2013	2014	2015
Number of Ignitions	25	40	19	15	24	5	136	440	139	606	391	54	518	191	300	93	31

#### Independent

Previous research has used various spatial measurements for IP modeling, and most variables come from three categories: socio-economic, biophysical, and topographic. Some relationships are well documented (e.g. the closer a pixel is to a road, the more likely an ignition is to occur), while others have been used sparingly (e.g. number of livestock in an area). The following independent variables from the literature were used as drivers of the IP model (Table 2).

**Table 2:** Independent variables and descriptive statistics for the study area.

2001 (2012)	Range	Mean	St. Dev.	Source	Data Type	Measurement
*Independent Variables*						
Distance to Road Way	0–4273.08 (0–1679.2)	323.72 (249.11)	411.59 (260.83)	U.S. Census Tiger Files	Continuous	Euclidean Distance in Meters from Road
Housing Density	0–391.249 (0–347.72)	0.72 (0.94)	3.18 (3.64)	U.S. Census	Continuous	Population per Hectare
Proportion Urban	0–100 (0–100)	19.88 (21.58)	31.34 (29.71)	HGAC	Continuous	Percent of Surrounding Cells Urban
Edge Density	0–178.698 (0–136.56)	29.46 (27.45)	23.61 (18.76)	LANDFIRE	Continuous	Urban Edge Density surrounding a pixel.
Land Cover	-	-	-	LANDFIRE	Discrete	13 fire behavior fuel models
Elevation	79–433	183.51	62.09	USGS National Elevation Dataset	Continuous	Meters relative to NAD83
Slope	0–47	2.47	3.31	Derived from USGS National Elevation Dataset	Continuous	Degree Slope

Distance to roads was measured using Euclidean distance and the Tiger 2010 road file from the U.S. Census Bureau. Roads that existed in 2000 were marked using aerial images, while new roads to 2010 were designated for the 2012 model. Housing density was calculated using census blocks normalized by hectares. Land cover uses 13 fire behavior fuel models (FBFM 13) which was provided by LANDFIRE (www.LANDFIRE.GOV). The elevation and slope datasets were also collected from LANDFIRE as products of the National Elevation Dataset (NED). The NED has a 900 m2 grain size and 1-meter vertical resolution. Other datasets may offer a higher resolution or grain size, the NED has the same grain size as the land cover and other inputs, which eliminated the need to upscale and resample. Proportion urban measures the percent of surrounding cells that are urban, and urban edge density. These variables were measured using the same 1920-m window size used above to categorize new development. This helped measure the amount of exposure each raster cell has from populated areas.

### Model validation

MaxEnt tests for sensitivity given a user-provided test dataset and background pixels using the Area Under the Curve (AUC) from the Receiver Operating Characteristic (ROC) analysis. The ROC measures each model’s sensitivity by assessing true positives and false positives between possible values. AUC values can range from 0–1 with AUC = 0.5 being completely random. Values are categorized as an excellent (>0.9) fit, a good (0.8–0.9) fit, and fair (0.7–0.8) fitting [48, 49]. Due to the limited sample size, the 2001 model used a smaller test dataset (n = 20) compared with the 2012 model (n = 99). The maximum AUC for an IP model is based on the area in which the presence sample covers as compared to the total study area [43]. It can be used to standardize the AUC and help determine if the model is maximizing its efficiency [50]. Additionally, using a Jackknife method MaxEnt provides the influence of each independent variable on the model's sensitivity. This output is shown as the sensitivity for each individual variable, and if that predictor variable was removed from the final model.

Goodness of fit is assessed through gain values [44], which describes how much variation in the model is explained by each variable if all others are held constant. Maximizing the gain value is similar to other optimization measures (e.g. AIC or BIC) and is one way of determining the optimal model [51].

Mean IP values were calculated surrounding development patches for the eleven categories: Urban Core, and 5 categories, infill, radial, clustered, linear, and isolated development for both Δt_1_ and Δt_2_ using a 30-meter buffer and associated with the development patch category. Differences in the probabilities were then tested using a two-way parametric ANOVA, followed by the Tukey *post hoc* assessment to determine the rankings of development type.

## Results

Qualitative assessment suggested that the recursive radial decisions, as well as the decision tree using patch area and PARA, were reasonable in categorizing development. Radial development occurred more often than all other development types combined. Clustered and isolated development were the least likely to occur.

**Fig 4:**
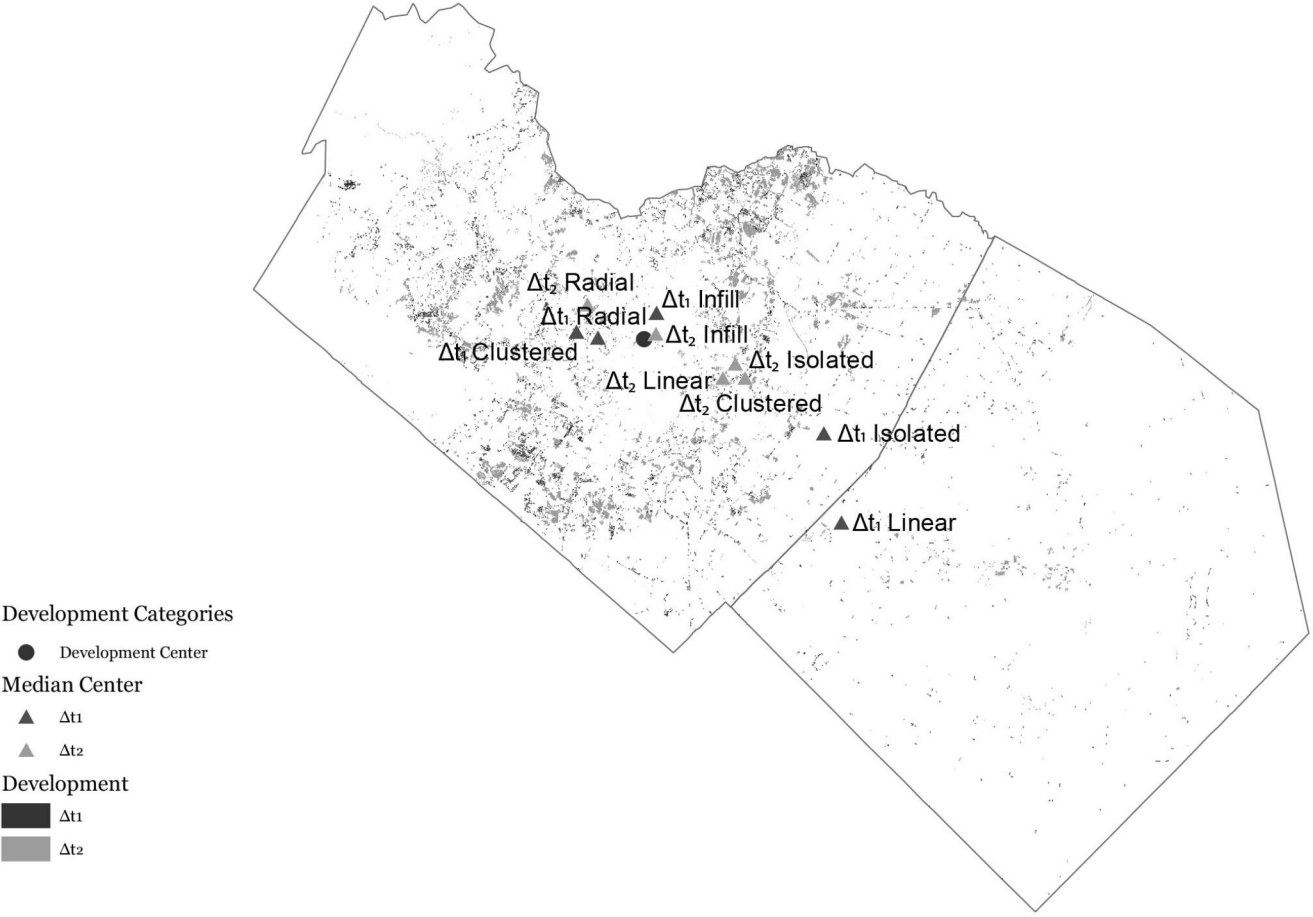
Generational development centers. Old generations tended to occur further from the urban core than the newer counter parts. This could potentially show evidence of a coalescing landscape.

Results showed that the center of all development occurred within the center of Travis County. Radial and infill occurred near this center of development, while the outlying development occurred further from the center. Δt1 development tended to occur further from the development center than Δt2 (Fig 4). Clustered development occurred to the western part of the study area, while isolated and linear development occurred further to the east. Within the Δt2, all three outlying development patterns occurred to the east.

Development pattern growth was similar between Δt_1_ and Δt_2_ (Table 3). More development occurred in Δt_2_ than Δt_1_ across all development patterns. This was most noticeable with clustered development. The primary development patterns were radial and infill; however, more infill was developed in Δt_2_ as the amount of growth was lower. This pattern was also seen with linear development which had the same amount of development.

**Table 3:** New development in ha and percentage of total development.

Development Type	Δt_1_		Δt_2_	
	Area (ha)	Percentage	Area (ha)	Percentage
Infill	1143	24.89%	1272	15.82%
Radial	3248	70.74%	5997	74.53%
Isolated	11	0.24%	83	1.04%
Clustered	61	1.35%	566	7.03%
Linear	127	2.78%	127	1.58%
Total	4592		8046	

**Fig 5:**
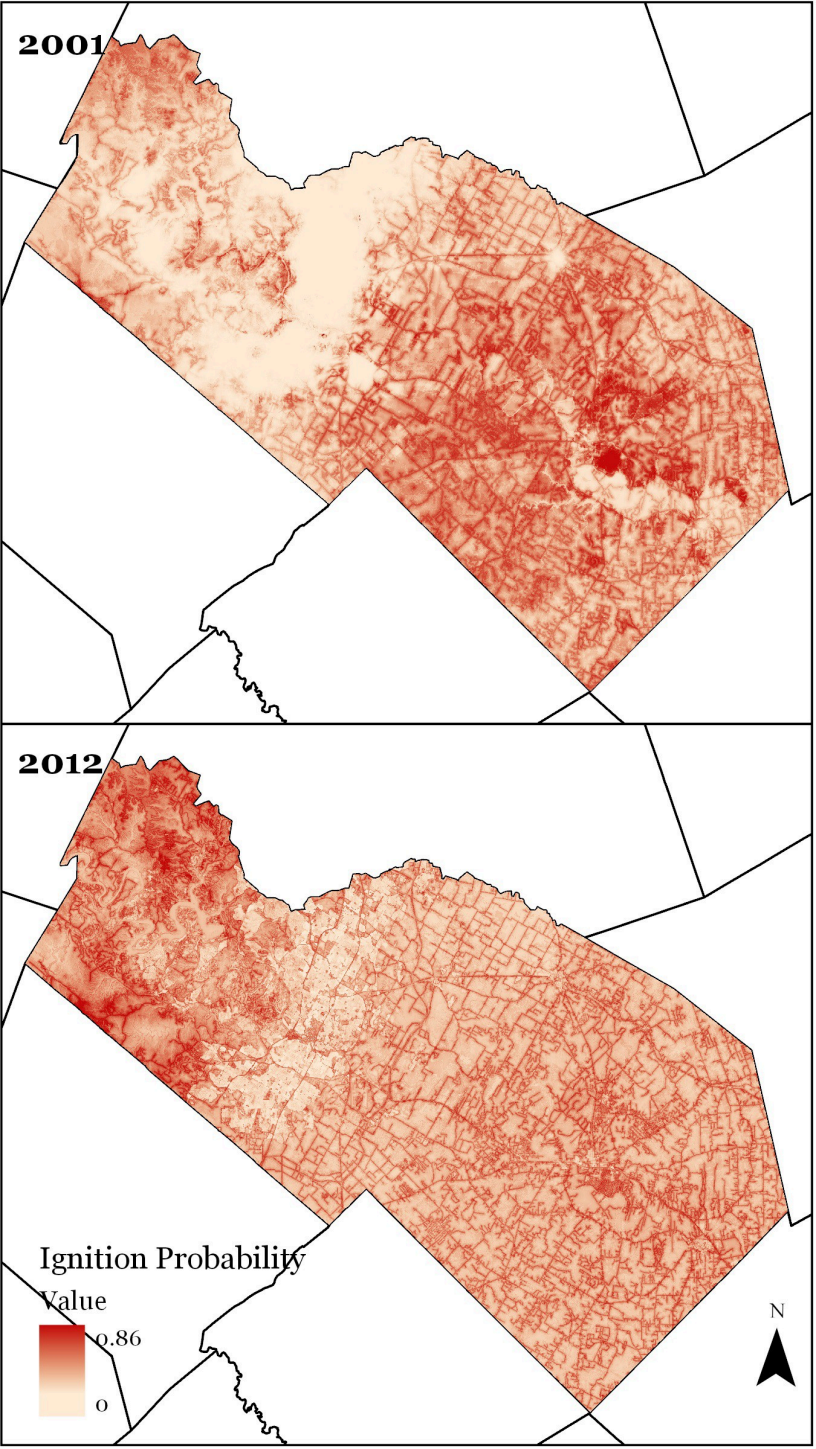
Variation in ignition probabilities across years. Ignition models were developed using MaxEnt software. Higher IP’s tended to occur in the Bastrop County, with the highest probabilities occurring in 2001. The basemap for this image uses census county data.

Across the landscape, mean ignitions probabilities were the same for both models (ip_2001_ = 0.28 and ip_2012_ = 0.28), while IP near development were higher in 2012 than 2001 across all development types (Fig 5). High ignition areas changed for the two models: in 2001 the high IP areas were located in southeastern Bastrop, while in the 2012 model higher IP’s were in northwestern Travis County. Roads were clearly a major driver of ignitions, as most of the road network is outlined by high IP rates, however the relationship appears clearer in 2012 than 2001.

The 2001 and 2012 sensitivities were not statistically different (AUC: 0.776–0.782, Table 4) when compared with its earlier counterpart (AUC: 0.771). The 2001 model (0.797) outperformed the 2012 models (0. 0.613) in gain values.

**Table 4:** IP Sensitivity and deviation from model AUC.

	Sensitivity			
	2001	2012		
AUC	0.782	0.7709		
Gain	0.797	0.613		
	Without Specified Variable		With Only Specified Variable	
	2001	2012	2001	2012
Distance to Roadway	-0.0333	-0.1553	-0.1152	-0.079
Housing Density	0.0086	-0.015	-0.1926	-0.1568
Elevation	0.0036	-0.0013	-0.1494	-0.2718
Land Cover	-0.0073	-0.0058	-0.2183	-0.2521
Slope	0.0008	-0.0132	-0.2601	-0.3207
Proportion Urban	-0.0326	-0.0107	-0.2301	-0.1867
Edge Density	-0.0165	-0.0048	-0.2446	-0.2334

### Sensitivity

Removing any single independent variable within the 2012 model decreased the overall sensitivity. Distance to roadway had the most impact, while removing elevation had the least impact. For the 2001 model, distance to roadways was still the most influential variable affecting the model's sensitivity (+0.033 to AUC), while housing density had the most negative effect (-0.0087 to AUC). Elevation was the only other variable that negatively influenced the model's sensitivity (-0.003 AUC when removed).

### Variable influence

**Fig 6:**
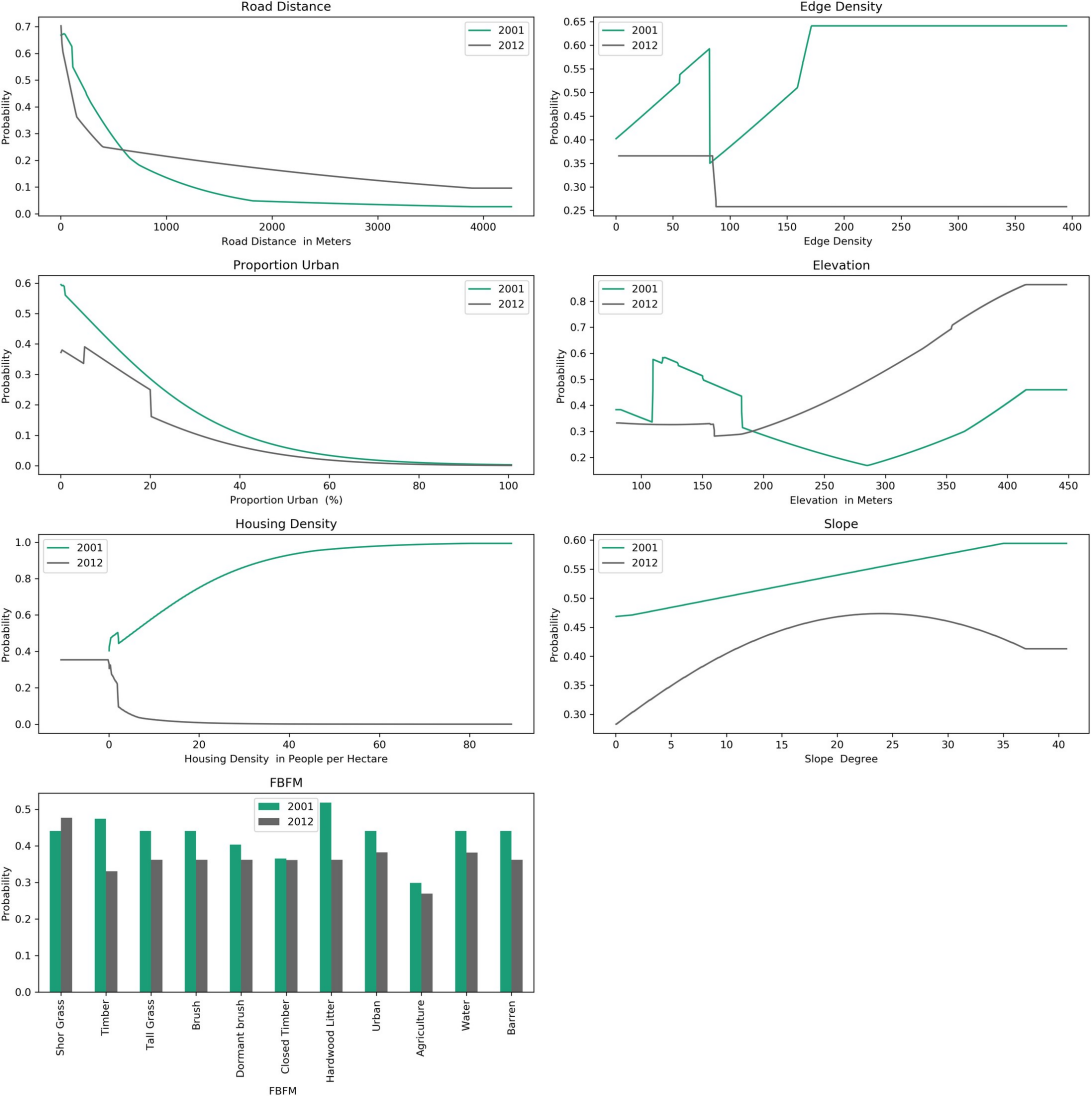
Variable influence on MaxEnt across years. Note the opposite effects of housing density and slope between years.

Holding all other variables constant, each independent variable initially increases the IP, and as the variable increases, IP decreases (Fig 6). The response curves for the 2001 model had several differences compared with the latter model. More specifically, the edge density and housing density variables within the 2001 and 2012 model had opposite effects on IP, where increases in both housing density and edge density increased IP in the 2001 model and decreased probability in the 2012 model. The slope in 2012 had a negative quadratic effect increasing up to 25 degrees and then decreasing, while the slope in the 2001 model increased IP over the range. Qualitatively, distance to roadways and proportion urban show exponential decay. The maximum IP for the variable distance to roadways occurs adjacent to roads.

### Statistical results

The two-way ANOVA for the 2001 and 2012 model (Table 5) demonstrated statistically significant differences among the 11 development types (*p*<0.000) and years (p<0.00). The total sample size between years was n = 95,390 for the eleven development categories.

**Table 5:** Two-way ANOVA, and Tukey’s *post hoc* analysis.

Number of obs = 95390 Residual = 3642.739022											
		Df	F	Prob>F							
Year		1	3715.87	0.00							
Development Type		10	756.33	0.00							
Year & Development Type		10	17.35	0.00							
Development Type	Δt_1_ Infill*	Δt_2_ Infill*	Core*	Δt_2_ Radial*	Δt_1_ Clustered*	Δt_1_ Radial*	Δt_2_ Clustered*	Δt_2_ Linear*	Δt_2_ Isolated*	Δt_1_ Linear*	Δt_1_ Isolated
2001 Mean	0.1424	0.1533	0.2061	0.2785	0.2802	0.2803	0.3597	0.3776	0.3845	0.4184	0.4578
2012 Mean	0.2180	0.200	0.2715	0.3705	0.4496	0.3817	0.4058	0.4306	0.4404	0.4703	0.5069
2001 Post Hoc Significance											
										
										
										
2012 Post Hoc Significance											
										
										
										

Note *denotes significance at 0.01 level. While underlines at the bottom of the table show development patterns which were not statistically different.

Most tests using the Tukey Kramer HSD showed a significance of at least *p*<0.01. For example, all development patterns, except for Δt_1_ isolated, increased in ignition probability between 2001 and 2012. In 2001, Δt_1_ infill had the lowest IP, while outlying development patterns of Δt_1_ linear and Δt_1_ isolated had the highest Ignition Probabilities. In the 2012 model the Δt_2_ Infill had the lowest IP, while Δt_1_ isolated remained the highest. Δt_1_ clustered and both Δt_1_ and Δt_2_ radial had the highest increases. Similar trends occur in the order of development pattern by Ignition Probability, with one exception, Δt_1_ clustered has the third highest ignition probability.

## Discussion

The trends found in this study coupled with the broader research focusing on development and IP suggest that policy makers should focus more on land use patterns in wildfire protection plans. The development patterns help explain the IP found on the landscape. The high amount of radial and infill development suggests that most development is occurring at a higher density and in the relatively lower IP areas near Travis County’s urban core. For example, Δt_2_ outlying development was often much closer to previously developed areas. Currently, the County Wildfire Protection Plans’ in Texas recommend (1) the spatial examination of the WUI, and (2) the setting of zoning standards to focus on structure level mitigation efforts [52]. These Wildfire Protection Plan recommendations fail to address better land use planning.

The results of this study support the hypothesis that Ignition Probabilities differ based on the nearest development type. Ignition Probabilities fell along a gradient from lower-to-higher probabilities for lateral development types of infill, radial, and outlying respectively. Despite adding new development between the 2001 and 2012 model, the Δt_2_ development categories could not be statistically differentiated from the Δt_1_ counterparts. This occurs despite locations of these development patterns being distinctly different within the study area. This risk gradient is similar to ones found in [34]. The response curve for the proportion urban variable adds support to the probability gradient finding, as both the 2001 and 2012 model reduce in probability as proportion urban increases. This gradient is likely due to a higher fuel to development ratio, where outlying development has a more surrounding fuel that could ignite. Researchers and historians have called for revisiting wildfire protection policies [53, 54], and focusing on the probability gradient could give distinct boundaries for where protection policies should shift. However, this gradient will need to be explored through wildfire behavior as well.

Moving beyond urbanization from remotely sensed urban land cover housing density response curves had opposite impacts for the 2001 and 2012 models. On average, housing density increases from 2001 to 2012. The flipping of the housing density response curve may be explained by areas of increased density becoming unburnable. This housing density change suggests that land use planners should focus on densifying area, which could also reduce overall suppression costs. While increasing the number of structures near a fire increases the cost to suppress that fire [33], suppression costs also vary by development pattern-individual homes disproportionately increasing the suppression costs compared to areas with clustered homes [55].

The other biophysical variables within the model were consistent with previous studies. For example, distance to roadways is often a primary driver, and the probabilities decay the further away from a road [47]. Slope is shown to have a positive effect on IP [30], but results indicate the relationship may be quadratic.

### Limitations and future research

One of the primary limitations of this research is driven by limited sample size. During 2001, local departments were not logging wildfire locations, resulting in lower sampling for 2001 and subsequent a subsample in 2012 to eliminate the local fire department ignitions. The sensitivity of the model with and without these ignitions was similar, however Ignition Probabilities were lower overall without the local fires. This could lead to bias of the spatial ignition pattern but is the more conservative model for the objectives. Future research would benefit from more comprehensive historical ignition data.

Second, the NLCD urban classifications had an 0.05% standard error in Texas [56], and the 30-meter resolution may omit homes and other structures. This may impact the findings, especially in the outlying developments. However, this was the best available data for the study. In addition, while this study focused on ignition probabilities, fire spread was not accounted for in this assessment. Future research should examine how the movement of wildfires on the landscape impact differing developments ignition probabilities.

## Conclusions

This paper built an Ignition Probability model to assess how IP varied with development changes. The model used the MaxEnt software along with several physical and environmental variables to predict historic ignition locations for two time periods (1999–2003 and 2010–2014) in Bastrop and Travis Counties. Using test subsets of the ignitions, sensitivities for the two models could not be statistically differentiated (AUC: 0.776–0.782). The results demonstrate that both physical factors and location influence wildfire ignition. Distance to Roadways and Proportion Urban were two primary factors influencing the models sensitivity in 2001, while Distance to Roadways and Slope were the two primary factors in 2012. A two-way ANOVA showed significance of IP between 2001 and 2012 and between lateral development type.

P*ost hoc* analysis during this study provides evidence for an Ignition gradient along lateral development. Those areas nearest previous urban development have lower probabilities, while outlying development patterns in the wildlands have higher probabilities. Proportion urban response curves in the MaxEnt model corroborates this ignition gradient. The gradient should inform future wildfire protection policies through land use planning and informed boundaries for mitigation practices. In addition, the Housing Density response curves suggests that land use planners should focus on densifying development to reduce IP.
